# Impact of Membrane Phosphoric Acid Doping Level on Transport Phenomena and Performance in High Temperature PEM Fuel Cells

**DOI:** 10.3390/membranes11110817

**Published:** 2021-10-26

**Authors:** Shian Li, Chengdong Peng, Qiuwan Shen, Chongyang Wang, Yuanzhe Cheng, Guogang Yang

**Affiliations:** Marine Engineering College, Dalian Maritime University, Dalian 116026, China; lishian@dlmu.edu.cn (S.L.); pengchengdong@dlmu.edu.cn (C.P.); wangchongyang@dlmu.edu.cn (C.W.); chengyuanzhe@dlmu.edu.cn (Y.C.); yanggg@dlmu.edu.cn (G.Y.)

**Keywords:** HT-PEMFCs, doping level, performance, transport characteristics

## Abstract

In this work, a three-dimensional mathematical model including the fluid flow, heat transfer, mass transfer, and charge transfer incorporating electrochemical reactions was developed and applied to investigate the transport phenomena and performance in high-temperature proton exchange membrane fuel cells (HT-PEMFCs) with a membrane phosphoric acid doping level of 5, 7, 9, 11. The cell performance is evaluated and compared in terms of the polarization curve. The distributions of temperature, oxygen mass fraction, water mass fraction, proton conductivity, and local current density of four cases are given and compared in detail. Results show that the overall performance and local transport characteristics are significantly affected by the membrane phosphoric acid doping level.

## 1. Introduction

Fuel cells are energy conversion devices where the chemical energy of a fuel is directly converted to electrical and thermal energy. Proton exchange membrane fuel cells (PEMFCs) can be used as a power source for various applications due to their high power density, high energy efficiency, and environmental friendliness [1,2]. The operating temperature of low-temperature proton exchange membrane fuel cells (LT-PEMFCs) is below <100 °C. The membrane of LT-PEMFCs must maintain a sufficient hydration level to ensure high proton conductivity. However, water flooding may occur inside fuel cells and then cause cell performance degradation. Therefore, water and thermal management strategies of LT-PEMFCs were extensively investigated [3,4,5]. In order to simplify the water and thermal management, high-temperature proton exchange membrane fuel cells (HT-PEMFCs) with operating temperature above 100 °C were proposed and studied. The advantages of HT-PEMFCs compared with LT-PEMFCs include high CO tolerance, fast reaction kinetics, and easy water management [6,7].

There are lots of studies related to the material and flow field design of HT-PEMFCs. The performance of fuel cells with different bipolar plate materials (aluminum, copper, and stainless steel) was studied numerically and experimentally by Wilberforce et al. [8]. It was reported that the fuel cell with the aluminum bipolar plate provides the best performance among the three different materials. Numerical studies were performed to investigate the performance of fuel cells with different channel to rib ratios [9]. The transport characteristics within fuel cells were analyzed and a maximum power density was observed with a ratio of 1.0. Bottomed-baffles were applied into the anode and cathode channels to improve cell performance [10]. The effect of the bottomed-baffle number on the polarization curves of fuel cells was systematically investigated. It was revealed that the maximum net cell power was provided by the fuel cell with five bottomed-baffles. Metal foams were used as a flow distributor of fuel cells to enhance cell performance [11]. It was reported that the current density improvement was achieved by the application of metal foams. Uniform distributions of species, temperature, and current density were also obtained. The transport phenomena in fuel cells with different flow field designs were numerically studied by Li et al. [12]. It was suggested that the flow field can be optimized to improve the cell performance and uniformity of the reactants inside fuel cells. Experimental studies were carried out to investigate the cell performance of fuel cells with serpentine and parallel channels [13]. Results showed that the fuel cell with serpentine channels provides better performance and higher pressure drop as well. A modified-serpentine flow field was designed and investigated numerically and experimentally by Singdeo et al. [14]. It was found that the uniformity of reactant and product distribution was enhanced compared with the serpentine flow field.

Numerical studies about the fuel cell components and operating conditions of HT-PEMFCs were also performed by researchers. The effects of membrane thickness and ionomer volume fraction in the catalyst layers on the performance were numerically analyzed by Mohanty et al. [15]. Their work confirmed that cell performance increases with decreasing membrane thickness. The cell performance is also strongly influenced by the ionomer volume fraction in the catalyst layers. The transport phenomena of fuel cells with/without microporous layers were numerically investigated and assessed by Nanadegani et al. [16]. The effect of adding microporous layers on heat and mass transfer processes was analyzed. The cell performance is significantly improved due to the presence of microporous layers. The mass transport impact on the cell performance was numerically studied by Das et al. [17]. The polarization curves of fuel cells under different inlet velocities, membrane thicknesses, membrane ionic conductivities, and porosities were obtained and compared. The effect of operating temperature on transport processes of heat, species, and charge inside fuel cells was numerically studied by Elden et al. [18]. The corresponding cell performance was also evaluated and compared.

The perfluorosulfonic acid (PFSA) membrane is used in LT-PEMFCs, while the polybenzimidazole (PBI) membrane is utilized in HT-PEMFCs. In order to obtain high proton conductivity, the PBI membrane is always doped with phosphoric acid, sulfuric acid, etc. [19,20,21]. Constant proton conductivities of the membrane are widely used in mathematical models to investigate the multi-physics phenomena and performance in HT-PEMFCs [10,15,17,21,22,23]. However, the membrane proton conductivity is a function of temperature and phosphoric acid doping level [24,25,26,27]. The transport processes and cell performance are greatly influenced by the membrane conductivity, which has a strong relationship with the phosphoric acid doping level. Therefore, it is necessary to perform a comprehensive investigation to examine the effect of membrane phosphoric acid doping level on local transport phenomena and overall performance in HT-PEMFCs. In the present work, a three-dimensional mathematical model including the fluid flow, heat transfer, mass transfer, charge transfer, and electrochemical reactions was developed and employed to study the transport characteristics and performance in HT-PEMFCs with different membrane phosphoric acid doping levels. The obtained results can improve the understanding of transport characteristics in HT-PEMFCs.

## 2. Model Description

### 2.1. Physical Model

**Figure 1:**
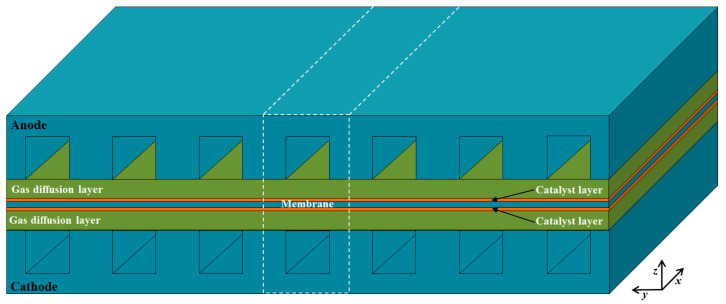
Schematic of a single fuel cell and the computational domain.

As shown in Figure 1, a single channel unit is selected as the computational domain, which includes the anode/cathode current collectors (CCs), gas flow channels (GFCs), gas diffusion layers (GDLs), catalyst layers (CLs), and membrane. The detailed geometric parameters of fuel cell components are given in Table 1. The operating temperature and pressure are 453.15 K and 1.0 atm, respectively. The reactants of hydrogen and air are provided at the anode and cathode inlet of GFCs with the co-flow arrangement, respectively. The assumptions made in the study are as follows: the fluid flow is laminar; ideal gas law is applied; the GDLs and CLs are isotropic and homogeneous; the membrane is impervious to reactant gases; contact resistances between different layers are neglected.

### 2.2. Governing Equations

The governing equations of mass, momentum, species, energy, charge are given as follows:

Mass equation:

(1) $$\nabla \cdot \left(\rho \vec{u}\right)=S_{\mathrm{mass}\;}$$

where ρ and $\vec{u}$ are the density and velocity, respectively. S_mass_ is the source term of the mass equation.

Momentum equation:

(2) $$\nabla \cdot \left(\rho \vec{u}\vec{u}\right)=\nabla \;\cdot \;\left(\mu \nabla \vec{u}\right)-\nabla P+S_{\mathrm{mom}}$$

where P and μ are the pressure and dynamic viscosity, respectively. S_mom_ is the source term of the momentum equation.

Species equation:

(3) $$\nabla \cdot \left({\rho \vec{u}Y}_{i}\right)=\nabla \;\cdot \;\left({\rho D}_{\mathrm{eff},i}\nabla Y_{i}\right)+S_{i}$$

where Y_i_ and D_eff,i_ are the mass fraction and effective diffusivity, respectively. S_i_ is the source term of the species equation.

(4) $$D_{\mathrm{eff},i}=\varepsilon^{1.5}D_{i,m}$$

Energy equation:

(5) $$\nabla \cdot \left({\rho C}_{p}\;\vec{u}T\right)=\nabla \;\cdot \;\left(k_{\mathrm{eff}}\nabla T\right)+S_{T}$$

where C_p_ and k_eff_ are the specific heat and effective thermal conductivity, respectively. S_T_ is the source term of the energy equation. The effective thermal conductivity can be determined by the following expression:

(6) $$k_{\mathrm{eff}}=\left(1-\varepsilon \right)k_{s}+{\varepsilon k}_{f}$$

where k_s_ is the thermal conductivity of the solid phase, and k_f_ is the thermal conductivity of the gas phase.

Charge equation:

(7) $$\nabla \cdot \left(\sigma_{\mathrm{eff},s}\nabla \phi_{s}\right)+S_{s}=0$$

(8) $$\nabla \cdot \left(\sigma_{\mathrm{eff},m}\nabla \phi_{m}\right)+S_{m}=0$$

where σ_eff,s_ is the effective electrical conductivity, σ_eff,m_ the effective proton conductivity, ϕ_s_ the electrical potential, ϕ_m_ the proton potential. S_s/m_ is the source term of the charge equation. The Butler–Volmer equation is applied to describe the hydrogen oxidation reaction and oxygen reduction reaction in the anode and cathode CLs, respectively.

The volumetric current density in the CLs can be determined by:

(9) $$j_{a}=i_{a}^{\mathrm{ref}}{\left(\frac{C_{H_{2}}}{C_{H_{2}}^{\mathrm{ref}}}\;\right)}^{0.5}\left[e^{\alpha_{a}{F\eta }_{a}/\mathrm{RT}}-e^{-\alpha_{c}{F\eta }_{a}/\mathrm{RT}}\right]$$

(10) $$j_{c}=i_{c}^{\mathrm{ref}}{\left(\frac{C_{O_{2}}}{C_{O_{2}}^{\mathrm{ref}}}\;\right)}^{1}\left[-e^{\alpha_{a}{F\eta }_{c}/\mathrm{RT}}+e^{-\alpha_{c}{F\eta }_{c}/\mathrm{RT}}\right]$$

The anode and cathode over-potentials are represented as:

(11) $$\eta_{a}=\phi_{s}-\phi_{m}$$

(12) $$\eta_{c}=\phi_{s}-\phi_{m}-V_{\mathrm{oc}}$$

The open circuit voltage can be determined by the following expression.

(13) $$V_{\mathrm{oc}}=1.1669-2.4\times 10^{-4}\left(T-373.15\right)$$

The membrane proton conductivity is a function of the temperature and phosphoric acid doping level [20,27]:

(14) $$\sigma_{m}=\frac{100}{T}\exp\left[8.0219-\left(\frac{2605.6-70.1\mathrm{DL}}{T}\right)\right]$$

where DL is the phosphoric acid doping level, and the effective electrical conductivity and proton conductivity of the GDLs and CLs are modified by the Bruggeman correlation:

(15) $$\sigma_{\mathrm{eff},s}={\left(1-\varepsilon_{\mathrm{GDL}}\right)}^{1.5}\sigma_{s}$$

(16) $$\sigma_{\mathrm{eff},s}={\left(1-\varepsilon_{\mathrm{CL}}-L_{i}\right)}^{1.5}\sigma_{s}$$

(17) $$\sigma_{\mathrm{eff},m}=L_{i}{}^{1.5}\sigma_{m}$$

The source terms of the above-mentioned governing equations are summarized in Table 2, and Table 3 gives the parameters used in the mathematical model.

### 2.3. Numerical Implementation

The implementation of the fuel cell mathematical model is performed in the software ANSYS FLUENT. The finite volume method is used for solving partial differential equations. The electron and proton transport are described by the equations defined by user-defined functions. At the inlet of the GFCs, the mass flow rate, temperature, and species mass fractions are prescribed. At the outlet of the GFCs, a pressure-outlet boundary condition is assigned. The anode and cathode mass flow rates are 7.117 × 10^−7^ kg/s and 2.073 × 10^−8^ kg/s, respectively. The detailed boundary conditions are illustrated in Table 4.

**Figure 2:**
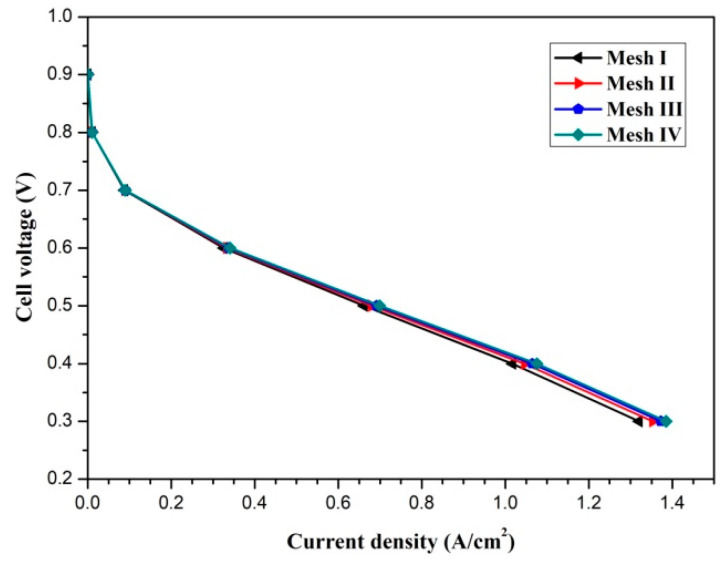
The mesh independence test.

The pressure and velocity fields are linked by the Semi-Implicit Method for Pressure Linked Equations (SIMPLE) algorithm. Four mesh systems (x × y × z), namely, mesh I (50 × 12 × 48), mesh II (75 × 16 × 64), mesh III (100 × 20 × 80), and mesh IV (125 × 24 × 96) are utilized to examine the mesh independence. The polarization curves of the fuel cell with different mesh numbers are presented in Figure 2. Mesh system III is selected to balance the accuracy and computational resources. The model validation was carried out and can be found in our previous study [11].

## 3. Results and Discussion

**Figure 3:**
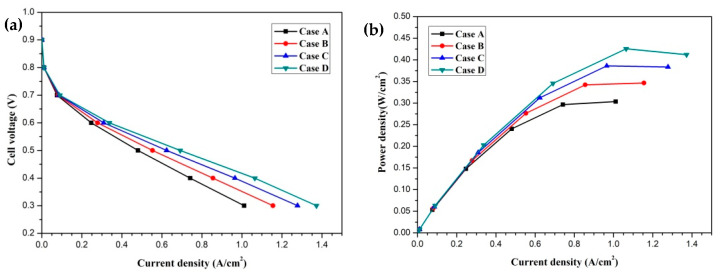
The cell performance of fuel cells with different membrane phosphoric acid doping levels (Case A: DL = 5; Case B: DL = 7; Case C: DL = 9; Case D: DL = 11): (**a**) cell voltage-current density curve; (**b**) power density-current density curve.

In this section, the transport characteristics and performance of HT-PEMFCs with different membrane phosphoric acid doping levels (DLs) are presented and compared in detail. Four cases are labeled as Case A, Case B, Case C, and Case D, which represent DL = 5, DL = 7, DL = 9, and DL = 11, respectively. The DL of the membrane is defined as the molar percentage of the acid (mol% H_3_PO_4_) per repeat unit of the PBI polymer [20]. The effect of the membrane phosphoric acid doping level on cell performance is given in Figure 3. The current density-cell voltage and current density-power density curves are presented and compared. The polarization curves of four cases are illustrated in Figure 3a. It is clearly seen that the cell performance is significantly affected by the DL and it is improved with an increase in DL. This is attributed to the decrease in the proton transport resistance. The proton conductivity is a function of temperature and DL. The proton conductivity is increased with increasing DL. This means that the proton transport process is enhanced and the ohmic loss is decreased. The current densities of four cases at the cell voltage 0.3 V are 1.011 A/cm^2^, 1.155 A/cm^2^, 1.278 A/cm^2^ and 1.372 A/cm^2^, respectively. The current density–power density curves are shown in Figure 3b. It can be observed that the power densities of four cases at the cell voltage 0.3 V are 0.303 W/cm^2^, 0.346 W/cm^2^, 0.383 W/cm^2^ and 0.412 W/cm^2^, respectively.

**Figure 4:**
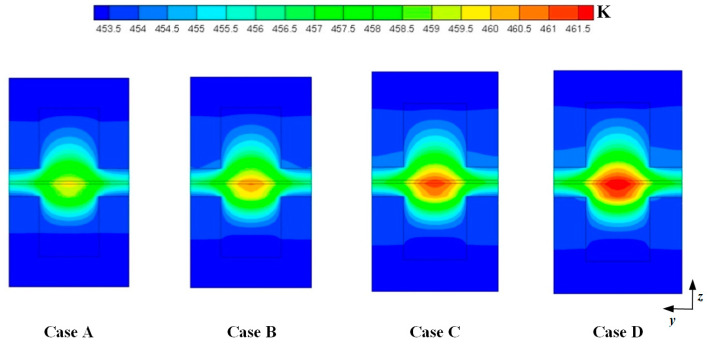
Temperature distributions at the middle plane of fuel cells (Case A: DL = 5; Case B: DL = 7; Case C: DL = 9; Case D: DL = 11).

Figure 4 shows the temperature distributions at the middle plane of fuel cells at the cell voltage 0.3 V. For the four cases, the maximum temperature is observed at the cathode side and the temperature under the channel is higher than that under the ribs. This is because the produced heat is easily dissipated through the ribs. It can also be seen that the maximum temperature is gradually increased when DL is increased from 5 to 11. This is because more heat can be generated when the current density is increased. As above mentioned, the current density is increased when DL is increased from 5 to 11. The values of the maximum temperature of four cases are 459.731 K, 460.578 K, 461.351 K, and 462.019 K, respectively.

**Figure 5:**
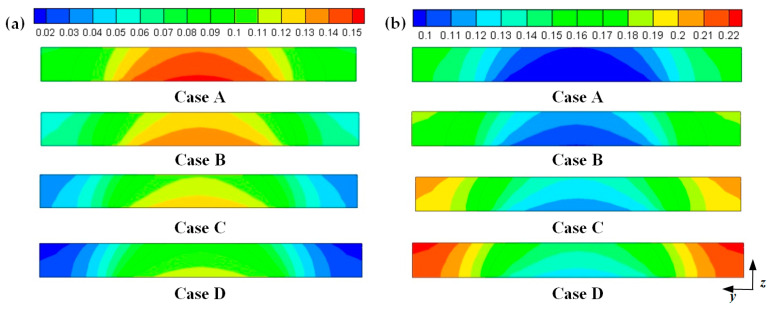
Species mass fraction at the middle plane of fuel cells (Case A: DL = 5; Case B: DL = 7; Case C: DL = 9; Case D: DL = 11): (**a**) oxygen mass fraction: (**b**) water mass fraction.

**Figure 6:**
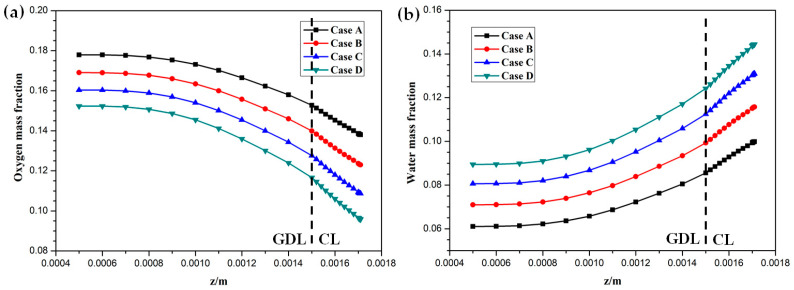
Species mass fraction profile distributions at y = 0.001 m (Case A: DL = 5; Case B: DL = 7; Case C: DL = 9; Case D: DL = 11): (**a**) oxygen mass fraction; (**b**) water mass fraction.

**Figure 7:**
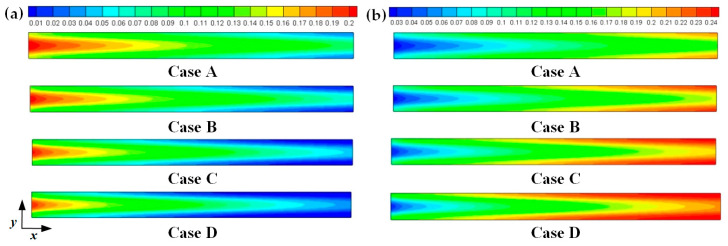
Species mass fraction at the cathode GDL and CL interface of fuel cells (Case A: DL = 5; Case B: DL = 7; Case C: DL = 9; Case D: DL = 11): (**a**) oxygen mass fraction; (**b**) water mass fraction.

**Figure 8:**
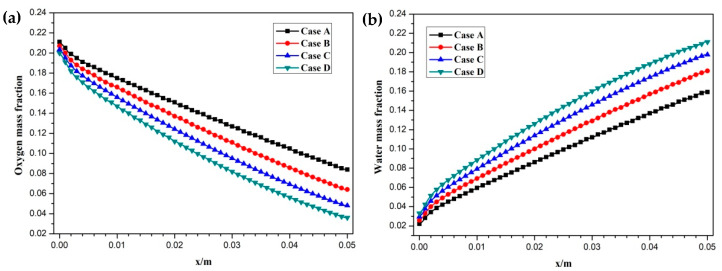
Species mass fraction profile distributions at y = 0.001 m (Case A: DL = 5; Case B: DL = 7; Case C: DL = 9; Case D: DL = 11): (**a**) oxygen mass fraction; (**b**) water mass fraction.

Hydrogen and oxygen are respectively transported from the anode and cathode GFCs and then consumed at the corresponding CLs. Meanwhile, water is produced at the cathode CL. The oxygen and water transport characteristics inside cathode CL and GDL of four cases at the cell voltage 0.3 V are shown in Figure 5 Figure 6 Figure 7 Figure 8. Figure 5 presents the oxygen and water mass fraction distributions at the middle plane of fuel cells. It is clearly seen that oxygen and water mass fraction is non-uniformly distributed due to the existence of ribs. The oxygen mass fraction under the channel region is greater than that under the rib regions, while the water mass fraction under the channel region is lower than that under the rib regions. This is because the species transport resistance is increased under the rib regions. The oxygen and water mass fraction profiles at the middle line of the above-mentioned planes are given in Figure 6. It is observed that the oxygen mass fraction gradually decreases, and the water mass fraction gradually increases from the GDL to the CL. This is attributed to the electrochemical reaction in the cathode CL. The oxygen mass fraction of four cases is decreased from 0.178 to 0.138, from 0.169 to 0.123, from 0.160 to 0.109, and from 0.152 to 0.096, respectively. And the corresponding water mass fraction is increased from 0.061 to 0.099, from 0.071 to 0.116, from 0.081 to 0.131, and from 0.089 to 0.144, respectively. Figure 7 shows the oxygen and water mass fraction distributions at the interface of GDL and CL. It can be seen that oxygen and water mass fraction is non-uniformly distributed along the flow direction due to the electrochemical reaction in the cathode CL. The oxygen and water mass fraction profiles at the middle line of the above-mentioned planes are plotted in Figure 8. It is observed that the oxygen mass fraction gradually decreases, and the water mass fraction gradually increases along the flow direction due to oxygen consumption and water production. The oxygen mass fraction of four cases is decreased from 0.211 to 0.084, from 0.207 to 0.064, from 0.203 to 0.048, and from 0.200 to 0.036, respectively. The corresponding water mass fraction is increased from 0.022 to 0.159, from 0.026 to 0.181, from 0.029 to 0.198, and from 0.033 to 0.211, respectively.

**Figure 9:**
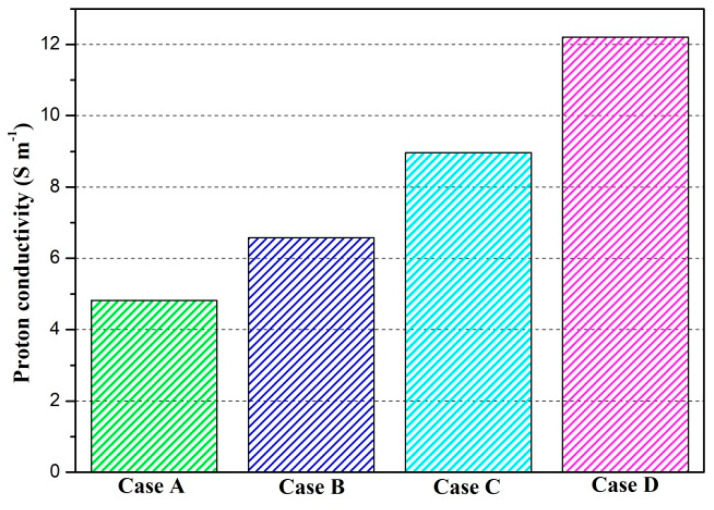
Proton conductivity of the membrane of four cases (Case A: DL = 5; Case B: DL = 7; Case C: DL = 9; Case D: DL = 11).

**Figure 10:**
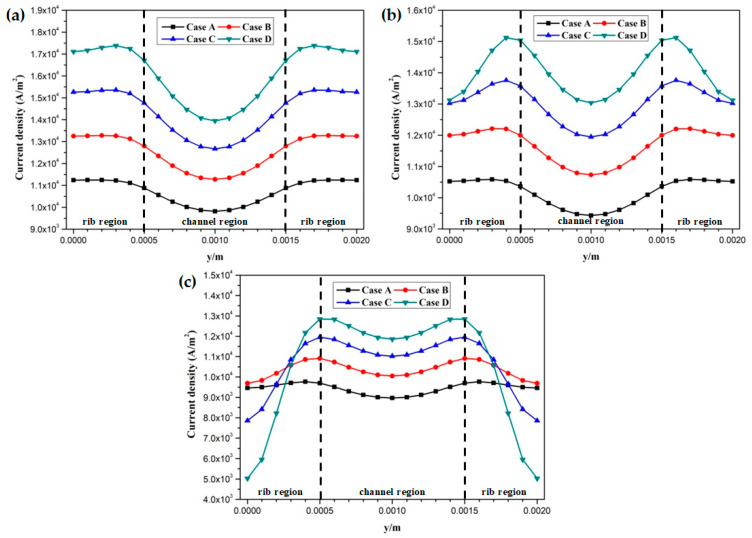
Local current density distribution of fuel cells at the middle plane of membrane (Case A: DL = 5; Case B: DL = 7; Case C: DL = 9; Case D: DL = 11): (**a**) x = 0.005 m; (**b**) x = 0.025 m; (**c**) x = 0.045 m.

The proton conductivities of the membrane of four cases at the cell voltage 0.3 V are given in Figure 9. It can be observed that the proton conductivity is gradually increased from Case A to Case D, and the proton conductivities of four cases are 4.82 S/m, 6.58 S/m, 8.96 S/m, and 12.20 S/m, respectively. Local current densities of four cases at the cell voltage 0.3 V are demonstrated in Figure 10. It is clearly observed each local current density profile is symmetrically distributed, and it is also gradually decreased along the flow direction. This is because the oxygen is gradually consumed along the flow direction and the molar concentration is also decreased. As shown in Figure 10a, the minimum current density is observed at the middle of the GFC region. The maximum current density is obtained by Case D, followed by Case C, Case B, and Case A. The local current density at the middle of the plane is given in Figure 10b. It can be seen that the current density of Case D and Case C sharply decreases at the rib regions, while that of Case B and Case A only slightly decreases at the same regions. In Figure 10c, Case D provides the maximum and minimum local current densities of four cases. This means that the current density of Case D is extremely non-uniformly distributed in comparison with other cases.

## 4. Conclusions

A three-dimensional mathematical model is established to investigate the performance and transport phenomena in HT-PEMFCs. The effect of membrane phosphoric acid doping level on polarization curves, distributions of temperature, species mass fraction, proton conductivity, and local current density are studied in detail. It is found that the current density at cell voltage 0.3 V is greatly improved from 1.011 A/cm^2^ to 1.372 A/cm^2^ when the doping level is increased from 5 to 11. The maximum temperature is observed at the cathode CL and increased with the increasing doping level. The distributions of oxygen and water mass fraction are also affected by the doping level. A higher water mass fraction and a lower oxygen mass fraction are observed with an increase in the doping level. The proton conductivity is also significantly increased with the increasing doping level. In addition, the trend and magnitude of local current density is strongly affected by the doping level.

## Figures and Tables

**Table 1:** Geometric parameters of the computational domain.

Parameter	Value	Unit
Cell length/width	50/2	mm
GFC height/width	1.0/1.0	mm
Anode/Cathode GDL thickness	0.2	mm
Anode/Cathode CL thickness	0.01	mm
Membrane thickness	0.05	mm

**Table 2:** Source terms of the governing equations.

Description	Units
$S_{\mathrm{mass}}=S_{H_{2}}$ (Anode CL) $S_{\mathrm{mass}}=S_{O_{2}}$ (Cathode CL) $S_{\mathrm{mass}}=S_{H_{2}O}$ (Cathode CL)	kg m^−3^ s^−1^
$S_{\mathrm{mom}}=-\frac{\mu }{K}\vec{u}$ (GDLs and CLs)	kg m^−2^ s^−2^
$S_{H_{2}}=-\frac{j_{a}}{2F}M_{H_{2}}$ (Anode CL)	kg m^−3^ s^−1^
$S_{O_{2}}=-\frac{j_{c}}{4F}M_{O_{2}}$ (Cathode CL)	kg m^−3^ s^−1^
$S_{H_{2}O}=\frac{j_{c}}{2F}M_{H_{2}O}\;$(Cathode CL)	kg m^−3^ s^−1^
$S_{T}=j_{a}\left|\eta_{a}\right|+\sigma_{\mathrm{eff},m}{\left\|\nabla \phi_{m}\right\|}^{2}+\sigma_{\mathrm{eff},s}\|\nabla \phi_{s}\|{}^{2}\;$(Anode CL) $S_{T}=j_{c}\left|\eta_{c}\right|-j_{c}\frac{{\mathrm{dV}}_{0}}{\mathrm{dT}}T+\sigma_{\mathrm{eff},m}{\left||\nabla \phi_{m}|\right|}^{2}+\sigma_{\mathrm{eff},s}{\left||\nabla \phi_{s}|\right|}^{2}$ (Cathode CL) $S_{T}=\sigma_{\mathrm{eff},m}{\left||\nabla \phi_{m}|\right|}^{2}$ (Membrane) $S_{T}=\sigma_{\mathrm{eff},s}{\left||\nabla \phi_{s}|\right|}^{2}$ (GDLs and CCs)	W m^−3^
$S_{s}=-j_{a}\;$(Anode CL) $S_{s}=+j_{c}\;$(Cathode CL)	A m^−3^
$S_{m}=+j_{a}\;$(Anode CL) $S_{m}=-j_{c}\;$(Cathode CL)	A m^−3^

**Table 3:** Parameters used in the mathematical model.

Parameter	Value	Units
Porosity of GDL/CL	0.6/0.4	-
Volume fraction of membrane in the CL	0.3	-
Anode/cathode reference exchange current density	1 × 10^9^/1 × 10^4^	A m^−3^
Anode transfer coefficient	0.5/1	-
Reference hydrogen concentration	40.88	mol m^−3^
Reference oxygen concentration	40.88	mol m^−3^
Thermal conductivity of CC/GDL/CL/membrane	20/1.2/1.5/0.95	W m^−1^ K^−1^
Electrical conductivity of CC/GDL/CL	10,000/1250/300	S m^−1^
Permeability of GDL/CL	1.18 × 10^−11^/2.36 × 10^−12^	m^2^
Hydrogen diffusivity	1.055 × 10^−4^ (T/333.15)^1.5^ (101,325/P)	m^2^ s^−1^
Oxygen diffusivity	2.652 × 10^−5^ (T/333.15)^1.5^ (101,325/P)	m^2^ s^−1^
Water diffusivity	2.982 × 10^−5^ (T/333.15)^1.5^ (101,325/P)	m^2^ s^−1^

**Table 4:** Parameters used in the mathematical model.

Description	Conditions	Value	Units
Anode terminal	ϕ_s_	0	V
Cathode terminal	ϕ_s_	V_cell_	V
Anode GFC inlet	Y	H_2_ = 1	-
	T	453.15	K
Cathode GFC inlet	Y	O_2_:N_2_ = 0.233:0.767	-
	T	453.15	K

## Data Availability

Data are contained within this study.
